# Intracellular amyloid toxicity induces oxytosis/ferroptosis regulated cell death

**DOI:** 10.1038/s41419-020-03020-9

**Published:** 2020-10-06

**Authors:** Ling Huang, Daniel B. McClatchy, Pamela Maher, Zhibin Liang, Jolene K. Diedrich, David Soriano-Castell, Joshua Goldberg, Maxim Shokhirev, John R. Yates, David Schubert, Antonio Currais

**Affiliations:** 1 The Razavi Newman Integrative Genomics and Bioinformatics Core, The Salk Institute for Biological Studies, 10010 N. Torrey Pines Rd., La Jolla, CA 92037 USA; 2 Department of Molecular Medicine and Neurobiology, The Scripps Research Institute, 10550 North Torrey Pines Road, La Jolla, CA 92037 USA; 3 Cellular Neurobiology Laboratory, The Salk Institute for Biological Studies, 10010 N. Torrey Pines Rd., La Jolla, CA 92037 USA

**Keywords:** Alzheimer's disease, Experimental models of disease

## Abstract

Amyloid beta (Aβ) accumulates within neurons in the brains of early stage Alzheimer’s disease (AD) patients. However, the mechanism underlying its toxicity remains unclear. Here, a triple omics approach was used to integrate transcriptomic, proteomic, and metabolomic data collected from a nerve cell model of the toxic intracellular aggregation of Aβ. It was found that intracellular Aβ induces profound changes in the omics landscape of nerve cells that are associated with a pro-inflammatory, metabolic reprogramming that predisposes cells to die via the oxytosis/ferroptosis regulated cell death pathway. Notably, the degenerative process included substantial alterations in glucose metabolism and mitochondrial bioenergetics. Our findings have implications for the understanding of the basic biology of proteotoxicity, aging, and AD as well as for the development of future therapeutic interventions designed to target the oxytosis/ferroptosis regulated cell death pathway in the AD brain.

## Introduction

A hallmark of Alzheimer’s disease (AD) is the accumulation of amyloid beta (Aβ) in extracellular plaques in the brains of patients. This observation has not only driven most of the basic research in the field, but also the development of therapeutics designed to diminish the plaque burden. To date, none of these therapies has succeeded in preventing or delaying the progression of AD^1^.

Although historically dismissed as being a technical artifact, a significant body of evidence now shows that Aβ accumulates inside neurons in the AD brain and that this accumulation is an early pathological event that precedes plaque formation itself^2–6^. Intraneuronal accumulation of Aβ has also been detected in nerve cell lines and transgenic mouse models of AD (reviewed in^2–6^).

This accumulation of Aβ can take place in a variety of subcellular compartments, including endosomes, multivesicular bodies, lysosomes, mitochondria, endoplasmic reticulum, Golgi apparatus, and cytosol^3,4^. Although toxic effects on specific physiological processes have been reported, such as synaptic disruption, inhibition of the ubiquitin-proteasome system, mitochondrial dysfunction, and activation of pro-inflammatory responses^6–8^, the exact molecular mechanism underlying the intraneuronal Aβ toxicity remains unclear.

Here, we addressed this gap in knowledge by using a systems biology approach to integrate transcriptomic, proteomic, and metabolomic data collected from a nerve cell model of toxic intracellular aggregation of Aβ. Using this approach, we found that intracellular Aβ causes nerve cells to die by the oxytosis/ferroptosis cell death program.

## Results

### Intracellular Aβ aggregation causes significant alterations in the transcriptome, proteome and metabolome of nerve cells

**Fig. 1:**
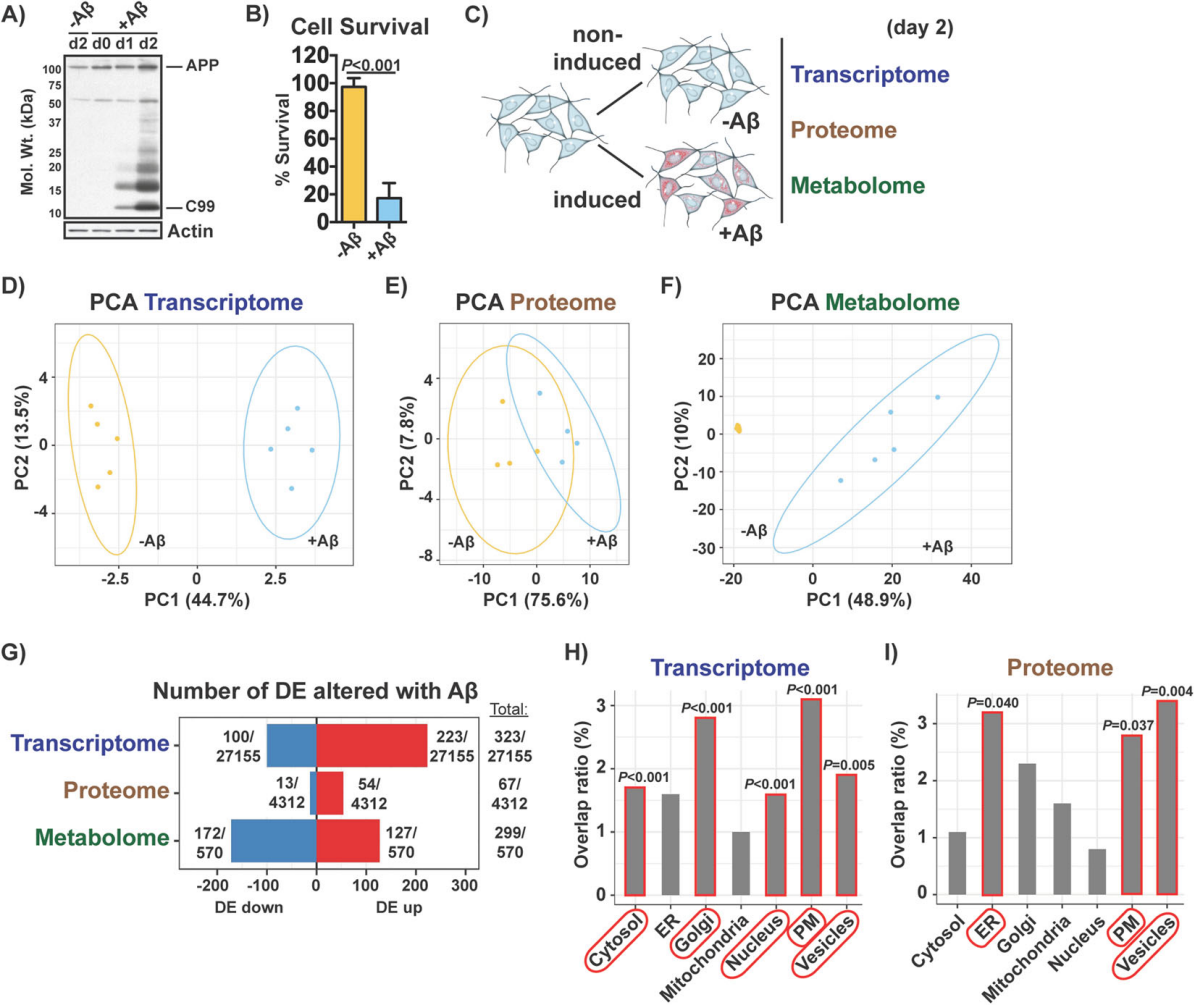
Global effects of intracellular Aβ on the transcriptome, proteome and metabolome of MC65 nerve cells. **a** Aβ aggregation in induced (+Aβ) MC65 nerve cells was confirmed by Western blotting at days 0, 1 and 2 and compared to non-induced (-Aβ) cells. Bars indicate C99 and full-length APP. **b** Cell survival was assayed on day 3. Unpaired two-tailed *t*-test (*n* = 3/group), data are mean ± SD. **c** Diagram illustrating the experimental approach. At day 2, non-induced (-Aβ) and induced (+Aβ) MC65 nerve cells were harvested for transcriptomic, proteomic and metabolomic analysis. PCA of the (**d**) top 500 most expressed genes (*n* = 5/group), (**e**) top 500 most expressed proteins (*n* = 4/group) and (**f**) total metabolites (*n* = 5/group). Ellipses show the 95% confidence interval of a t-distribution. Fold change levels and respective adjusted *P* values for all parameters can be found in supplementary Tables S1, S2 and S3. **g** Number of DE genes, DE proteins and DE metabolites upregulated (up) and downregulated (down) with Aβ. Number of total genes, proteins and metabolites detected/measured is indicated as the denominator. DE cutoff for the three parameters was FDR < 0.05 and absolute logFC > 0.5. Distribution of the (**h**) DE genes and (**i**) DE proteins per major subcellular compartment according to the Human Protein Atlas^14^. Overlap ratio is the number of DE genes or DE proteins found altered per total genes or proteins described to be present in a given compartment. *P* values calculated by 1000 bootstrap are indicated.

In order to identify the molecular mechanism(s) by which intracellular Aβ is toxic to neurons, we carried out a comprehensive multi-omics analysis to study the transcriptome, proteome, and metabolome of MC65 nerve cells in the absence and presence of intracellular Aβ toxicity. The MC65 is a human nerve cell line that expresses the C99 fragment of the amyloid precursor protein (APP) under the control of a tetracycline-sensitive promoter^9^. The parent cell line is SK-N-MC from a human neuroblastoma, and it has an electrically excitable membrane typical of neurons^10^. When tetracycline is withdrawn, MC65 nerve cells express C99 which is then converted to Aβ by γ-secretase and the cells die within 3 days due to Aβ accumulation and aggregation within cells (Fig. 1a, b)^8^. An increase in tau phosphorylation is observed in these cells upon induction of Aβ^11^. There is no extracellular Aβ in MC65 culture media^9,12,13^, and in the presence of γ-secretase inhibitors, cells express C99 but Aβ is not generated and cells do not die^8^. For the omics experiments, cells with tetracycline and no tetracycline were harvested 2 days after induction, prior to cell death. Samples were then processed for whole RNA sequencing, global protein mass spectrometry, and large-scale metabolomics (Fig. 1c).

Principal component analysis (PCA) of the top 500 most variable genes, top 500 most variable proteins and total metabolites identified a clear separation between the non-induced (-Aβ) and induced (+Aβ) cell groups for each of the three molecular parameters (Fig. 1d–f). This separation was clearest with the transcriptomic and metabolomic data (Fig. 1d, f). The effects of Aβ were most pronounced on the metabolome, where the greatest separation between treatments was observed (Fig. 1f).

A total of 323 out of 27155 genes (~1.2%), 67 out of 4312 proteins (~1.6%) and 299 out of 570 metabolites (~52.5%) examined were differentially expressed (DE) in the presence of Aβ (Fig. 1g). While the majority of the DE metabolites were decreased, most of the DE genes and DE proteins were elevated (Fig. 1g).

We then asked in which organelles these changes were taking place. To address this, we mapped the DE genes and DE proteins to the major subcellular compartments according to the Human Protein Atlas^14^. We found that the DE genes were associated with proteins that localize in a variety of compartments, but preferentially to the Golgi (*P* < 0.001) and plasma membrane (*P* < 0.001) (Fig. 1h). The DE proteins were mostly associated with the endoplasmic reticulum (*P* = 0.040), plasma membrane (*P* = 0.037) and vesicles (*P* = 0.004) (Fig. 1i).

These data indicate that the cell death associated with intracellular Aβ is preceded by significant changes in the transcriptome, proteome, and metabolome of MC65 nerve cells that take place in multiple cellular compartments.

### Pathway analysis identifies a pro-inflammatory, metabolic reprogramming associated with oxytosis/ferroptosis during Aβ toxicity

**Fig. 2:**
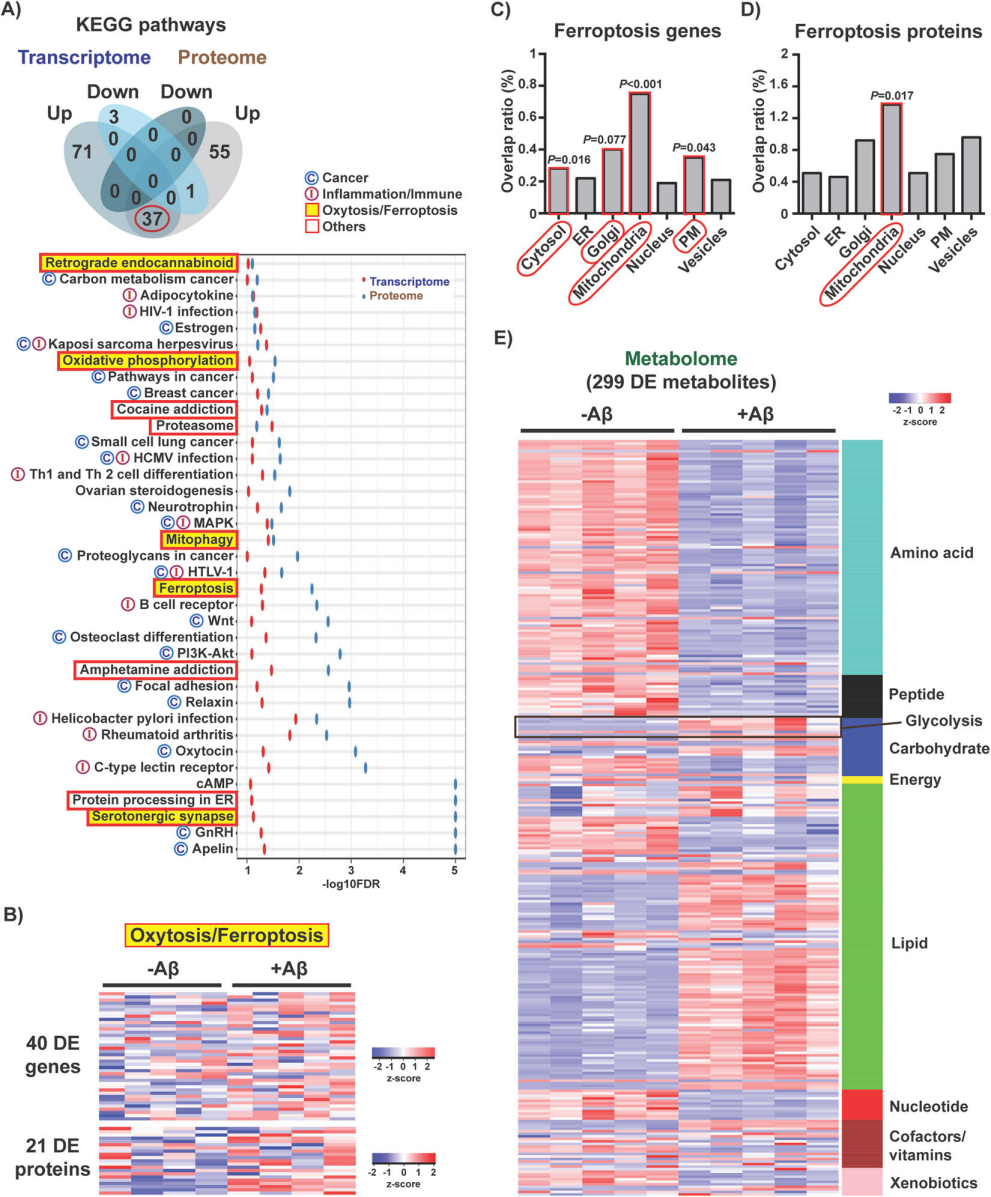
Identification of the cellular pathways associated with Aβ toxicity. **a** Venn diagram illustrating shared and uniquely affected KEGG pathways identified by GSEA that were upregulated (up) or downregulated (down) with Aβ. The red circle indicates the 37 KEGG pathways that were commonly altered in both transcriptomic and proteomic data. A list of those 37 pathways ordered by averaged enrichment score (-log10FDR) is shown. Pathways are grouped by biological process (cancer, inflammation/immune, oxytosis/ferroptosis and others). **b** Heatmap of the fold expression of the 40 DE genes (*n* = 5/group) and 21 DE proteins (*n* = 4/group) associated with the KEGG pathway ferroptosis. *z*-score = row-wise normalized log-transformed FPKM values for genes or batch-corrected log-transformed normalized average spectrum values for proteins. Distribution of the (**c**) 40 DE genes and (**d**) 21 DE proteins associated with ferroptosis per major subcellular compartment according to the Human Protein Atlas^14^. Overlap ratio is the number of DE genes or DE proteins found altered per total genes or proteins described to be present in a given compartment. *P* values calculated by 1000 bootstrap are indicated. **e** Heatmap of the 299 DE metabolites organized by biological class (*n* = 5/group). z-score = row-wise normalized Metabolon normalized imputed spectrum values.

To identify the specific molecular pathways underlying the alterations observed in the omics data, we first carried out Gene Set Enrichment Analysis (GSEA) with both the transcriptomic and proteomic data (metabolites have no gene equivalent). By overlapping the KEGG pathways obtained from both datasets by GSEA, 37 common pathways were identified that were all activated by Aβ and could be grouped into three main categories (Fig. 2a). Two of these categories included pathways that were associated with cancer (C) and inflammatory/immune (I) responses, which were frequently overlapping (Fig. 2a), in accordance with our previous reports^8^. The third group was associated with ferroptosis (highlighted in yellow) and processes that are known to be disrupted by ferroptosis, including mitochondrial function (KEGG terms: oxidative phosphorylation, mitophagy) and calcium signaling (KEGG terms: retrograde endocannabinoid, serotonergic synapse) (Fig. 2a)^15^. Other pathways included the proteasome and protein processing in the endoplasmic reticulum (Fig. 2a), likely reflecting the stress directly caused by Aβ accumulation.

Ferroptosis is a form of non-apoptotic regulated cell death characterized by the iron-dependent accumulation of lipid peroxides^16,17^ that extensively overlaps mechanistically with oxytosis, a previously described non-apoptotic cell death program characterized by glutathione (GSH) depletion, dysregulated production of reactive oxygen species (ROS) and consequent lipid peroxidation^15,18–20^. Therefore, oxytosis and ferroptosis have been proposed to be the same process and will henceforth be referred as oxytosis/ferroptosis^15^. Because cancer cells are more susceptible to die via oxytosis/ferroptosis^16,17^, we reasoned that the three KEGG categories identified could have common ground in oxytosis/ferroptosis. For this reason, we focused on further studying this degenerative process.

Heatmap representations of the DE genes and DE proteins associated with the ferroptosis KEGG pathway show an overall increase in their expression with Aβ (Fig. 2b). To investigate whether these genes and proteins were associated with specific organelles, we mapped them to the subcellular compartments described in the Human Protein Atlas^14^. The data show that both the ferroptotic DE genes and DE proteins were predominantly associated with the mitochondria (Fig. 2c, d).

At the level of the metabolome, heatmap visualization of the 299 DE metabolites organized by biological class shows a clear decrease in amino acid and nucleotide metabolism accompanied by an overall increase in lipid species in MC65 nerve cells expressing Aβ (Fig. 2e).

**Fig. 3:**
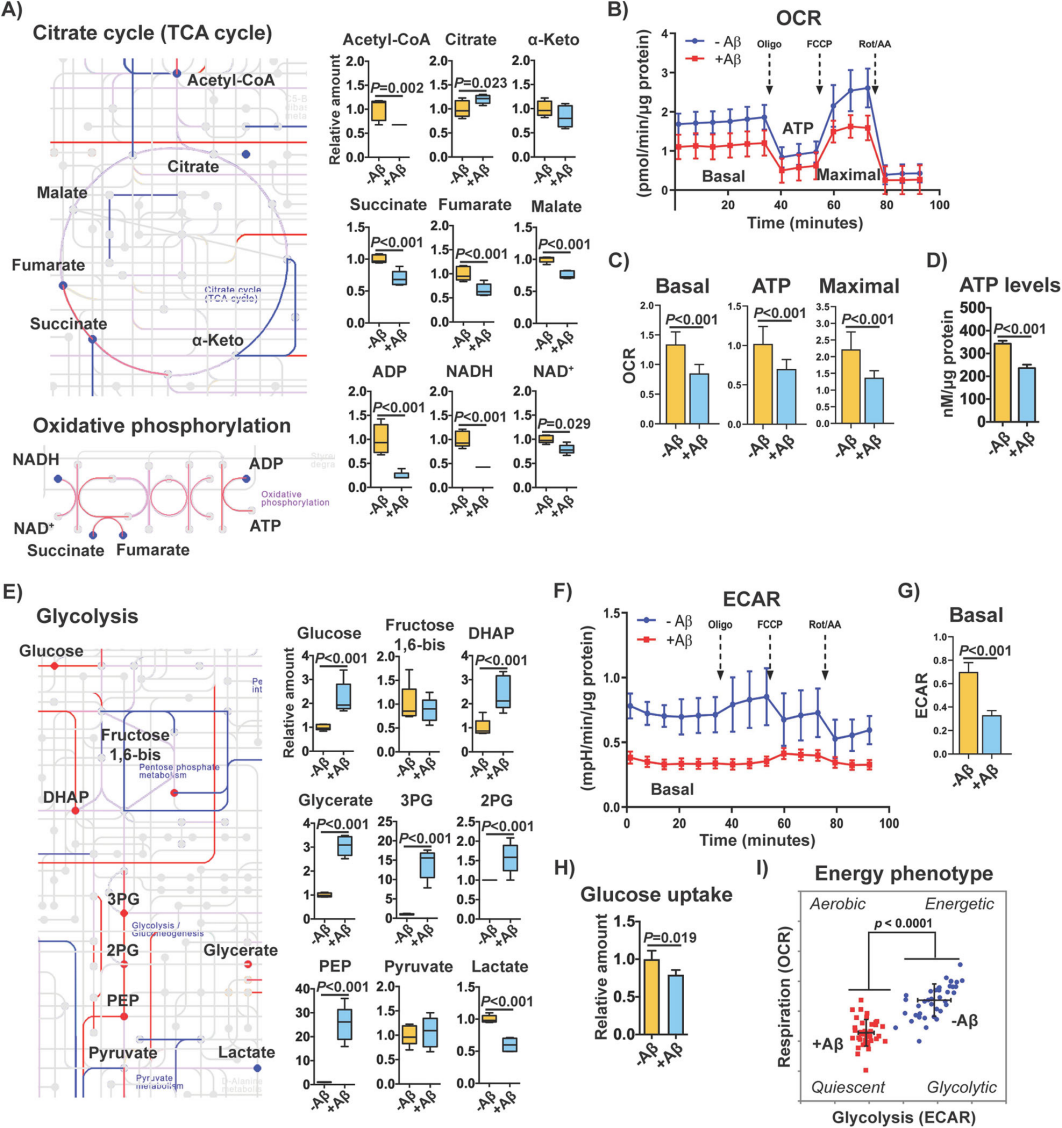
Effects of intracellular Aβ on central carbohydrate metabolism. **a** Extracts from the biochemical chart in Fig. S1 depicting the TCA cycle and oxidative phosphorylation biochemical pathways. Upregulated genes and proteins are shown in red lines, and downregulated genes and proteins in blue lines. The color is purple when genes and proteins did not show the same direction of change. Upregulated and downregulated metabolites are indicated in red and blue circles, respectively. DE cutoff for the three parameters was FDR < 0.05 and absolute logFC > 0.5. Graphs with levels of the different metabolites measured are also shown (*n* = 5/group). The metabolites citrate, malate and NAD^+^ had an absolute logFC < 0.5 but are also represented. **b** Mitochondrial oxygen consumption rates (OCR) in non-induced (-Aβ) and induced (+Aβ) MC65 nerve cells after 2 days. **c** The graphs for respective basal respiration, ATP production and maximal respiration are indicated. Unpaired two-tailed *t*-test (*n* = 40/group). **d** Total ATP levels in non-induced (-Aβ) and induced (+Aβ) MC65 nerve cells. Unpaired two-tailed *t*-test (*n* = 3/group). **e** Extract from Fig. S1 depicting the glycolysis biochemical pathway, with respective changes in genes, proteins and metabolites. DE cutoff for the three parameters was FDR < 0.05 and absolute logFC > 0.5. Graphs with levels of the glycolytic metabolites measured are shown (*n* = 5/group). **f** Extracellular acidification rate (ECAR) in MC65 nerve cells after 2 days of Aβ induction. **g** Basal ECAR. Unpaired two-tailed *t*-test (*n* = 40/group). **h** Glucose uptake at day 2 after Aβ induction. Unpaired two-tailed *t*-test (*n* = 4/group). **i** Cellular energy phenotype, determined by plotting the basal levels of OCR versus ECAR. Aerobic: cells rely predominantly in mitochondrial respiration. Glycolytic: cells utilize predominantly glycolysis. Energetic: cells utilize both metabolic pathways. Quiescent: cells are not very energetic via either metabolic pathway. All data are mean ± SD.

We then integrated all of the transcriptomic, metabolomic, and proteomic data into the master KEGG metabolic map (KEGG: hsa01100) (Fig. S1). This chart highlights the key biochemical pathways affected by Aβ and provides information on the relationship between the three molecular parameters. Changes in amino acid metabolism, glycolysis, lipid metabolism, nucleotide metabolism, oxidative phosphorylation (OXPHOS), and the tricarboxylic acid (TCA) cycle were observed in cells expressing Aβ (Fig. S1). In support of the transcriptomic and proteomic data, decreases in multiple TCA cycle and OXPHOS metabolites were detected, including acetyl-CoA, succinate, fumarate, malate, ADP, NADH and NAD^+^ (Fig. 3a). These changes were associated with a reduction in not only mitochondrial basal respiration, ATP production and maximal respiratory capacity (Fig. 3b, c), but also total cellular ATP levels (Fig. 3d). These data indicate that mitochondrial bioenergetics is severely disrupted by Aβ and support the evidence from Fig. 2a identifying mitochondrial-related pathways underlying its toxicity.

In contrast to decreased levels of TCA metabolites, the levels of multiple glycolytic intermediates were elevated, including glucose, dihydroxyacetone phosphate, glycerate, 3-phosphoglycerate, 2-phosphoglycerate, and phosphoenolpyruvate (Fig. 3e). Assessment of the extracellular acidification rate (ECAR), an indirect measure of extracellular lactate produced by glycolysis, showed low levels of basal ECAR (Fig. 3f, g), in accordance with the reduced levels of intracellular lactate (Fig. 3e). Decreases in glucose uptake (Fig. 3h) were also detected, suggesting that the increase in the levels of glycolytic intermediates may be a consequence of impaired conversion of pyruvate into both acetyl-CoA in the mitochondria and lactate in the cytosol. Along with the deficits in mitochondrial respiration, the decrease in glucose uptake and glycolytic flux due to Aβ toxicity are indicative of a hypometabolic state (Fig. 3i).

Altogether, these data demonstrate that Aβ causes a severe metabolic reprogramming associated with mitochondrial dysfunction, a well-known consequence of oxytosis/ferroptosis^15^.

### Aβ toxicity induces the main hallmarks of oxytosis/ferroptosis in nerve cells

**Fig. 4:**
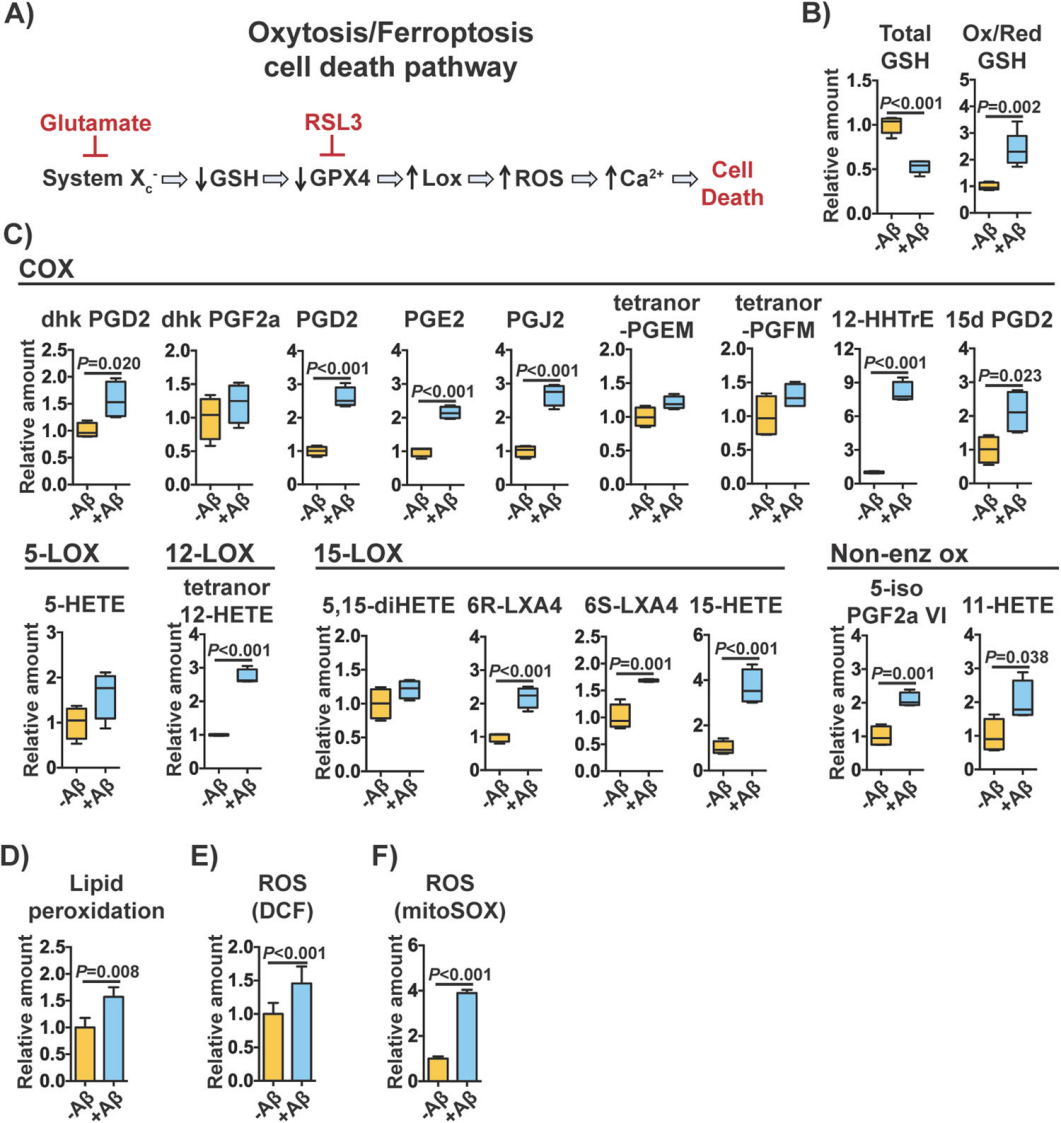
Main features of oxytosis/ferroptosis in MC65 nerve cells exposed to Aβ toxicity. **a** Diagram illustrating the molecular cascade characteristic of the oxytosis/ferroptosis cell death program. **b** Levels of total and oxidized/reduced GSH in MC65 nerve cells after 2 days of Aβ induction (*n* = 5/group). **c** Levels of eicosanoids derived from enzymatic (COX, 5-LOX, 12-LOX, 15-LOX) and non-enzymatic oxidation of AA measured at day 2. 13,14-dihydro-15-keto-prostaglandin D_2_ (dhk PGD2); 13,14-dihydro-15-keto-prostaglandin F_2_ alpha (dhk PGF2a); prostaglandin D_2_ (PGD2); prostaglandin E_2_ (PGE2); prostaglandin J_2_ (PGJ2); tetranor-prostaglandin EM (tetranor-PGEM); tetranor-prostaglandin EM (tetranor-PGFM); 12-hydroxyheptadecatrenoic acid (12-HHTrE); 15-deoxy-delta 12,14-prostaglandins D^2^ (15d PGD2); (5-HETE); tetranor-12-hydroxyeicosatetraenoic acid (tetranor 12-HETE); 5,15-dihydroxyeicosatetraenoic acid (5,15-diHETE); 6R-lipoxin A_4_ (6R-LXA4); 6S-lipoxin A_4_ (6S-LXA4); 15-hydroxyeicosatetraenoic acid (15-HETE); prostaglandin F_2_ alpha VI (5-iso PGF2aVI); 11-hydroxyeicosatetraenoic acid (11-HETE). Unpaired two-tailed *t*-test (*n* = 4/group). **d** Global lipid peroxidation at day 2. Unpaired two-tailed *t*-test (*n* = 3–4/group). **e** Whole cell ROS production at day 2. Unpaired two-tailed *t*-test (*n* = 11/group). **f** Mitochondrial superoxide production day 2. Unpaired two-tailed *t*-test (*n* = 3/group). All data are mean ± SD.

In order to further examine the role of oxytosis/ferroptosis in Aβ-induced proteotoxicity, as suggested by the pathway analysis (Fig. 2a), we analyzed the main features of this cell death program at day 2 after induction of Aβ in MC65 nerve cells. At the molecular level, oxytosis/ferroptosis can be triggered by glutamate that inhibits cystine uptake via system x_c_^-^ and subsequently depletes intracellular GSH^15,18,20^ (Fig. 4a). This leads to inhibition of the GSH-dependent enzyme GSH peroxidase 4 (GPX4) and activation of lipoxygenases (LOXs)^21^. GPX4 can also be directly inhibited by RSL3. In both cases, ROS and lipid hydroperoxides are generated, which potentiate intracellular calcium influx through store-operated calcium channels and cell death (Fig. 4a)^15,18,20^.

We first observed that the levels of GSH were indeed reduced in MC65 nerve cells with Aβ and that the ratio of oxidized/reduced GSH was increased (Fig. 4b), indicative of oxidative stress. We next measured the levels of several oxidized species of arachidonic acid (AA), a major fatty acid constituent of cell membrane phospholipids targeted by lipid peroxidation. Both non-enzymatically and enzymatically (COX, 5-LOX, 12-LOX and 15-LOX) oxidized species of AA were significantly elevated in the presence of Aβ (Fig. 4c). The total level of intracellular lipid peroxides was also increased, as detected by flow cytometry with the probe bodipy 581/591 C11 (Fig. 4d). Finally, we assessed the levels of ROS. A significant increase in ROS production was detected with dichlorofluorescein (DCF) (Fig. 4e) and with mitoSOX (Fig. 4f), a probe that is specific for ROS generated in the mitochondria. These data show that Aβ induces the major hallmarks of the oxytosis/ferroptosis pathway in MC65 nerve cells prior to cell death.

### Oxytosis/ferroptosis is the cell death program underlying intracellular Aβ toxicity

**Fig. 5:**
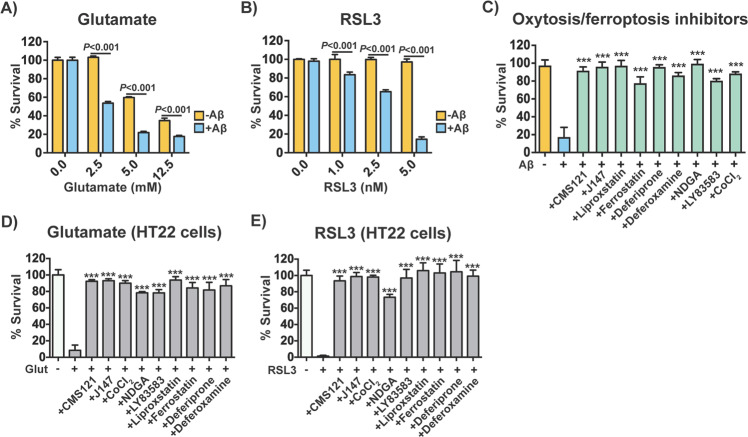
Induction and inhibition of oxytosis/ferroptosis in MC65 nerve cells. Susceptibility of non-induced and induced MC65 nerve cells to (**a**) glutamate and (**b**) RSL3 assessed at day 2 after induction of Aβ. Two-way repeated measures ANOVA and Tukey post hoc corrected *t*-test (*n* = 3/group). **c** Effects of oxytosis/ferroptosis inhibitors in non-induced and induced MC65 nerve cells on day 3. The inhibitors tested included: CMS121 (1 μM), J147 (1 μM), liproxstatin (10 μM), ferrostatin (10 μM), deferiprone (75 μM), deferoxamine (20 μM), NDGA (0.5 μM), LY83583 (1 μM), and CoCl_2_ (25 μM). Two-way repeated measures ANOVA and Tukey post hoc corrected *t*-test (*n* = 6/group). Effects of oxytosis/ferroptosis inhibitors in HT22 nerve cells exposed to (**d**) glutamate (10 mM) and (**e**) RSL3 (100 nM). The oxytosis/ferroptosis inhibitors included: CMS121 (1 μM), J147 (1 μM), liproxstatin (5 μM), ferrostatin (5 μM), deferiprone (75 μM), deferoxamine (50 μM), NDGA (10 μM), LY83583 (0.5 μM), and CoCl_2_ (100 μM). Two-way repeated measures ANOVA and Tukey post hoc corrected *t*-test (*n* = 6/group). ***P < 0.001. All data are mean ± SD.

To investigate whether activation of oxytosis/ferroptosis in MC65 nerve cells upon Aβ induction plays a role in cell death, we tested the effects of multiple, well-described modulators of oxytosis/ferroptosis in our model. By day 2, MC65 nerve cells that produce toxic Aβ were significantly more sensitive than non-induced cells to the oxytosis/ferroptosis inducers glutamate (Fig. 5a) and RSL3 (Fig. 5b). On the other hand, two AD drug candidates CMS121 and J147^22^ that were developed on the basis of inhibiting oxytosis/ferroptosis, also prevented Aβ-mediated cell death, as assessed at day 3 (Fig. 5c). Likewise, inhibition with the anti-oxytotic/ferroptotic compounds liproxstatin and ferrostatin, as well as the iron-chelators deferiprone and deferoxamine, also prevented cell death caused by Aβ (Fig. 5c). In addition, reducing lipid peroxidation with the pan-LOX inhibitor nordihydroguaiaretic acid (NDGA) and preventing intracellular influx of calcium with the guanylate cyclase inhibitor LY83583 or the calcium channel antagonist cobalt chloride (CoCl_2_), all protected MC65 nerve cells from Aβ toxicity (Fig. 5c). We confirmed that, as expected, these inhibitors were protective against glutamate and RSL3 in HT22 nerve cells as well (Fig. 5d, e). In summary, together, our data identify and confirm that oxytosis/ferroptosis is the molecular mechanism responsible for the cell death induced by intracellular Aβ in MC65 nerve cells.

## Discussion

Intraneuronal Aβ is observed early in the development of AD, and some believe it to be the primary event in the disease process^2–6^. We report here that intracellular accumulation of Aβ causes nerve cell death via oxytosis/ferroptosis. To our knowledge, this is the first time that three different omic analyses have been combined to understand a biological disease phenotype. Pathway analysis of all the data identified oxytosis/ferroptosis as a key feature of Aβ in MC65 nerve cells, which was further confirmed mechanistically. Although oxytosis/ferroptosis has been reasonably well characterized in culture, much less clear is the role that it plays in human disease. This is because less attention has been paid to how the pathological changes underlying oxytosis/ferroptosis may build up gradually over the course of time and thus contribute to disease. However, there is evidence that the same features that define oxytosis/ferroptosis in culture can also be detected in the aging and dementia brain. The most prominent include decreases in the levels of GSH^23^, iron accumulation^24^, and increased lipid peroxidation^25^. While most AD drug discovery has been designed to target the extracellular Aβ plaques, none has succeeded in producing an effective treatment, and new therapeutic alternatives are urgently needed^1^. As such, understanding the oxytosis/ferroptosis pathway in the context of human disease cannot be overstated, for it will identify new drug targets.

In a number of publications, we have now demonstrated that several AD drug candidates developed on the basis of being anti-oxytotic/ferroptotic also protect in mouse models of AD and aging where little or no neurodegeneration occurs^22,26–28^. This suggests that the oxytosis/ferroptosis cell death pathway is important to aging and AD and that it is a process that manifests itself over a lengthy amount of time before the cells die, thereby offering a window for therapeutic intervention. Given that aging and a number of neurodegenerative diseases are associated with intracellular protein aggregation (such as AD, Parkinson’s disease and Huntington’s disease), our findings have broad implications for diseases where proteotoxicity is a central event.

Integration of the data from our transcriptomics, proteomics, and metabolomics analysis showed that oxytosis/ferroptosis induced by Aβ in MC65 nerve cells was strongly associated with pathways that are activated during inflammation and cancer. This is important because cancer cells are more susceptible to die via oxytosis/ferroptosis^16,17^. The metabolic reprogramming that leads to increased glycolysis and lipid biosynthesis is a defining feature of what is known as the Warburg effect, and is a hallmark of both cancer and inflammation^29^. That our KEGG pathway analysis identified cancer and inflammation as main features of Aβ toxicity, while in fact the cells were in a condition of hypometabolism, suggests that cells were attempting to activate pathways involved in adaptation to conditions of energetic insufficiency caused by Aβ. Similar to cancer cells, these metabolic changes might predispose MC65 nerve cells to oxytosis/ferroptosis. In addition, we have previously shown that Aβ induces a strong pro-inflammatory response in MC65 nerve cells with cytokine release via, at least in part, the activation of the receptor for advanced glycation endproducts (RAGE)^8^. The cytokines further exacerbated the Aβ aggregation and toxicity. As such, damage-associated molecular patterns, which can be generated from dysfunctional mitochondria, may also contribute to Aβ-induced oxytosis/ferroptosis via RAGE binding and activation.

The role of mitochondria in oxytosis/ferroptosis has been investigated, and it is widely recognized that mitochondria are negatively affected by oxytosis/ferroptosis^18,30,31,15,20^. In our experiments, mitochondrial dysfunction in MC65 nerve cells mediated by Aβ was indicated by: (1) pathway analysis; (2) omics integration into the KEGG biochemical chart; (3) decreases in mitochondrial metabolites; (4) impaired mitochondrial bioenergetics; and (5) generation of mitochondrial ROS. These data are extremely important because mitochondrial dysfunction may be a critical event that bridges aging to AD and has therapeutic value^22,32^.

One of the mitochondrial metabolites that was significantly decreased was acetyl-CoA. Acetyl-CoA is a central metabolite linking glycolysis to the TCA cycle, lipid synthesis/oxidation, and metabolism of branched chain amino acids^33^. We have shown recently that acetyl-CoA levels were also decreased in HT22 nerve cells exposed to oxytosis/ferroptosis and in a mouse model of accelerated aging (SAMP8)^22^. Importantly, the two AD drug candidates CMS121 and J147 slowed the progression of aging in SAMP8 mice via a common mechanism that targeted acetyl-CoA metabolism and was associated with the maintenance of mitochondrial function with aging^22^. Therefore, the potential role of acetyl-CoA loss in oxytosis/ferroptosis warrants further investigation.

It is not clear why the levels of many amino acids and their related metabolites were lowered by Aβ toxicity. In cancer cells, an abundant supply of amino acids is required to sustain their proliferative drive via the biosynthesis of new proteins, lipids and regulation of redox homeostasis^34,35^. We did observe considerably more proteins (and gene transcripts) that were elevated by Aβ in MC65 nerve cells than those that were lowered. We also detected large increases in overall lipid species, which could be a consequence of the activation of cancer-related pathways^34,35^.

Although the mitochondria were critically affected by Aβ, our data show that oxytosis/ferroptosis may have broad functional consequences in MC65 nerve cells involving other organelles. Not only has Aβ been reported to accumulate in multiple compartments within neurons^3,4^, but oxytosis/ferroptosis causes the oxidation of lipids in the plasma membrane as well as other membrane bound organelles that eventually leads to cells’ demise^17^.

The activation of oxytosis/ferroptosis by Aβ may be happening by one or more different mechanisms. One possibility is through endoplasmic reticulum stress. Accumulation of intracellular Aβ can induce endoplasmic reticulum stress responses^13,36^, which can cause a decrease in GSH levels because GSH is required by the enzymes in the endoplasmic reticulum responsible for the folding and disulfide bond formation of newly synthesized proteins^37^. In MC65 cells, there is a large depletion of GSH following the expression of Aβ^38^, which our data confirmed. In addition, ROS within the endoplasmic reticulum is increased during conditions of stress caused by the elevated expression of misfolded proteins^39^. As such, when stressed, the endoplasmic reticulum significantly contributes both to oxidative stress and the depletion of reduced GSH^39^. Another possibility is through mitochondrial dysfunction, which can also contribute to the generation of ROS and decreases in GSH. Mitochondrial metabolism and ROS can be altered as a consequence of endoplasmic reticulum stress via Ca^2+^ signaling^40^, but also directly by intracellular Aβ, which can be imported into mitochondria and negatively impact their function^41^. Therefore, oxidative stress from the endoplasmic reticulum and/or mitochondria may contribute to oxytosis/ferroptosis in MC65 nerve cells. This increase in oxidative stress could be directly responsible for the activation of LOXs^42,43^ seen in the MC65 nerve cells as indicated by the generation of multiple LOX eicosanoids as well as increased global lipid peroxidation and neuroprotection with a pan-LOX inhibitor. Importantly, impaired LOX activity has been reported in post-mortem brains of AD patients as well as transgenic mouse models of AD^42,44^. It should also be noted that increases in tau phosphorylation have been identified in MC65 nerve cells upon induction of Aβ^11^. The contribution of tau phosphorylation to oxytosis/ferroptosis in our system as well as other models of tau pathology that lead to neuronal degeneration warrant further investigation.

In summary, our investigation identified and confirmed oxytosis/ferroptosis as the cell death program induced by the toxic intracellular accumulation of Aβ in MC65 nerve cells. Aβ accumulates within neurons in the brains of AD patients, and is likely an early pathological event that precedes plaque formation itself^2–6^. While in MC65 nerve cells this process is accelerated via the abrupt induction of intracellular Aβ aggregation, in humans the slow accumulation of Aβ inside neurons may induce a prolonged state of oxytosis/ferroptosis that disrupts neuronal basic functions and also contributes to additional toxic responses in the brain such as those from glia. Therefore, this manuscript has substantial implications for the understanding of basic AD pathology and for the development of future therapies that may be designed to target the oxytosis/ferroptosis pathway in the AD brain.

## Methods

All reagents were obtained from Sigma-Aldrich (St. Louis, MO, USA), unless otherwise stated.

### Intracellular amyloid toxicity in MC65 nerve cells

The MC65 nerve cells were obtained from Dr. L. W. Jin (UC Davis). The induction of intracellular amyloid toxicity in MC65 nerve cells was performed exactly as described^8^. MC65 nerve cells express the C99 fragment of the amyloid precursor protein (APP) under the control of a tetracycline-mediated promoter. Cells are grown in Dulbecco’s Modified Medium plus 10% fetal calf serum and 2 μg/ml tetracycline. The removal of tetracycline induces C99 synthesis and C99 is cleaved to Aβ. Usually, by day 3 cells in the absence of tetracycline are dead, while control, uninduced cells remain viable. Cell viability was determined by the 3-(4, 5-dimethylthiazolyl-2)-2,5- diphenyltetrazolium bromide (MTT) assay as previously described^45^. In all cases, cells in the dishes were examined microscopically before the addition of the MTT reagent to ensure that any positive results in the MTT assay are not an artifact due to interaction of the extracts with the assay chemistry. The data are presented as viability relative to controls plus tetracycline. Cells are routinely tested for mycoplasma and no contamination has been found.

### SDS-PAGE and immunoblotting

Western blots were carried out as described previously^27^. The primary antibodies used were: beta amyloid 6E10 (#803004, BioLegend); HRP-conjugated rabbit anti-actin (#5125, Cell Signaling Technology). Horseradish peroxidase-conjugated secondary goat anti-mouse (BioRad) antibody was used.

### Whole transcriptome analysis

RNA was isolated from MC65 nerve cells using the RNeasy Plus Universal mini kit (Qiagen). RNA-Seq libraries were prepared using the Illumina TruSeq Stranded mRNA Sample Prep Kit according to the manufacturers instructions. Briefly, poly-A RNA was selected using poly dT-beads. mRNA was then fragmented and reverse transcribed. cDNA was end- repaired, adenylated and ligated with Illumina adapters with indexes. Adapter-ligated cDNA was then amplified. Libraries were pooled and sequenced single-end 50 base-pair (bp) on the Illumina HiSeq 2500 platform.

RNA-Seq reads were mapped by STAR [v2.5.3a, ref: 10.1093/bioinformatics/bts635. pmid:23104886] to the hg19 reference genome with default parameters and flag “–outFilterIntronMotifs RemoveNoncanonical”. Homer [v4.9.1, ref: PMID: 20513432; http://homer.ucsd.edu/homer/] commands “makeTagDirectory” and “analyzeRepeats” were used to quantify gene expression by counting uniquely mapped reads across all exons of RefSeq genes. Mitochondrial gene reads were quantified using STAR with flag “–quantMode GeneCounts” to the 10X cell ranger hg19 v1.2.0 STAR index because they were not included in Homer annotation. Differential expression analysis was performed using DESeq2 [v1.21.22, https://genomebiology.biomedcentral.com/articles/10.1186/s13059-014-0550-8]. Genes with log fold-change (logFC) > 0.5 and false discovery rate (FDR) < 0.05 were identified as significantly changed.

### Proteomic analysis

#### Sample preparation for MS analysis

Cell pellets were resuspended in lysis buffer (10 mM Tris pH 8, 150 mM NaCl, 1 mM EDTA, 0.1% SDS, 1% Triton, 1% Sodium Deoxycholate, 2.5 mM MgCl_2_, 50 units/ml Benzonase (Sigma) with protease and phosphatases Inhibitor tablets (Roche). After overnight incubation on a rotator at 4 °C, samples were centrifuged for 30 min at 17,000 × *g*. The protein in the supernatant was quantified with the Pierce BCA protein assay kit (Thermo Fisher). 100 μg of sample were precipitated with Methanol/Chloroform. The dried pellets were resuspended in 8 M urea, reduced with 5 mM TCEP for 20 min at 37 °C, and then alkylated with 10 mM Chloroiodoacetamide for 20 min at room temperature. The samples were diluted with 200 mM TEAB for a final urea concentration of 2 M. Trypsin was added (1:50 of enzyme to substrate) and incubated overnight on a 37 °C shaker. The digested peptides were labeled using a 10-plex TMT kit (Thermo Fisher, Lot#TI273434). Each biological replicate was labeled with a different tag. The remaining two TMT tags of the 10-plex were pooled from all the samples. The labeled peptides from each MS dataset were combined then fractionated to 8 fractions using the High pH Reversed-Phase Peptide Fractionation Kit (Thermo Fisher).

Normalized average spectrum counts for proteome data were log-transformed and tested by limma [v.3.34.9, https://academic.oup.com/nar/article/43/7/e47/2414268] to identify the significantly DE proteins induced by Aβ with consideration of batch information. A pseudo count of 5 was added to each value before log-transformation. Proteins with logFC > 0.5 and FDR < 0.05 were identified as significantly changed.

#### MS analysis

An Easy-1000 UPLC coupled to an Orbitrap Lumos Tribid (Thermo Fisher) was used for MS analysis. Samples were eluted on a column self-packed with BEH (Ethylene BridgedHybrid) (Waters 100 μm inner diameter × 1.7 μm × 20 mm) using a 1–30% gradient of solvent B for 160 min, 30–90% for 60 min, and 90% for 20 min at a 200 μL/minflow rate. Two blanks were run after each biological replicate to avoid carry-over effects. The Orbitrap Lumos was operated in data-dependent acquisition mode using the Multinotch MS3 method through the XCalibur software. Survey scan mass spectra were acquired in a positive ion mode in the 400–1500 m/zrange with the resolution set to 120,000 (fwhm) and AGC target of 4 × 10^5^ on the Orbitrap. The ten most intense ions per survey scan containing 2–7charges were selected for CID fragmentation, and the resulting fragments (MS2) were analyzed in the ion trap in the 400–120 m/z range. Dynamic exclusion was employed within 10 s to prevent repetitive selection of the same peptide. The ten most intense MS2 fragments were selected for HCD fragmentation (MS3), and the resulting fragments were detected in the Orbitrap in the 120–500 m/zrange with the resolution set to 15,000 (fwhm) and AGC target of 10^5^.

#### Data processing

Raw files were extracted into ms1, ms2, and ms3 files using Raw Converter 1.1.0.19 (http://fields.scripps.edu/rawconv/). For each MS dataset, data from each fraction were combined prior to database searching. The MS2 files were interpreted by Prolucid and results were filtered, sorted, and displayed using the DTA Select 2 program using a decoy database strategy filtering with a 10 ppm mass accuracy and the final protein false discovery rate was <1%^46,47^. Mass shifts of 229.1629 on lysine/N-terminus and 57.02146 on cysteine were searched as static modifications. Using the MS3 files, the TMT tags were quantitated with Census^48^. For each tag, the intensity of a given reporter ion was normalized by the sum of the total intensity of all reporter ions. The MS1 files were employed to analyze the “purity” of identified peptide to avoid contamination of the reporter ion quantitation by co-eluting peptides. A purity filter of 0.6 was employed. The raw files for the MS analysis were deposited at ftp://MSV000085059@massive.ucsd.edu.

### Large-scale metabolome analysis

Metabolite measurement and analysis was conducted at Metabolon as previously described^27,49^. For statistical analyses and data display, any missing values were assumed to be below the limits of detection and imputed with the compound minimum (minimum value imputation).

### ATP measurements

ATP levels were measured by bioluminescence according to the manufacturer’s instructions (Molecular Probes, A22066).

### Glucose uptake

Uptake of glucose from media was determined by bioluminescence using the glucose uptake-glo^TM^ assay (Promega, J1341), following the manufacturer’s instructions.

### Seahorse XF analysis

Cellular oxygen consumption rate (OCR) and ECAR were assayed with a XF Cell Mito Stress Test (Agilent Technologies) using a Seahorse XFe96 Extracellular Flux Analyzer (Seahorse Bioscience, North Billerica, MA), according to the manufacture’s guidelines. Briefly, XF base DMEM medium was supplemented with 10 mM glucose, 1 mM pyruvate and 2 mM L-glutamine, at pH 7.4. Inhibitors were used at the following concentrations: 1.5 µM oligomycin, 2 µM FCCP, and 0.5 µM of a mixture of rotenone and antimycin A. Analyses were conducted using Wave software and XF Report Generators (Agilent Technologies). The sensor cartridge for XFe analyzer was hydrated at 37 °C 1 day before the experiment. OCR and ECAR data were normalized for total protein/well. Each condition was analyzed in 8–40 wells.

For MC65 nerve cells, non-induced and induced cells after 2 days were resuspended in Seahorse XF DMEM assay medium and plated at 40,000 cells/well in Agilent Seahorse XFe96 microplates. The microplates were centrifuged at 200 *g* for 5 min at room temperature and incubated for 1 h at 37 °C prior to the XF Cell Mito Stress Test.

### Eicosanoid analysis

All solvents were of chromatography purity. Eicosanoids used for primary standards in standard curves as well as their deuterated analogs were from Cayman Chemicals and Biomol (Enzo Life Science, Farmingdale, NY, USA). For extraction, 900 μL of conditioned culture media from MC65 cells were supplemented with a cocktail consisting of 26 deuterated internal standards. Samples were then purified by solid phase extraction on Strata-X columns (Phenomenex, Torrance, CA, USA) following the activation procedure provided by the distributor. Samples were eluted with 1 mL of 100% methanol, the eluent was dried under vacuum and dissolved in 50 μL of buffer A consisting of 60/40/0.02 water/acetonitrile/acetic acid = 60/40/0.02 (v/v/v) and immediately used for analysis.

### Lipid peroxidation measurements

Bodipy 581/591 C11 was used to quantify total lipid peroxidation in MC65 nerve cells by flow cytometry. Briefly, non-induced and induced cells at day 2 were incubated with 1 μM bodipy 581/591 C11 for 30 min, gently scraped and lipid peroxidation was measured by the loss of fluorescence (λ excitation = 530 nm, λ emission = 590 nm). 1 μg/ml DAPI was included to gate out any damaged cells.

### ROS measurements

For ROS detection, MC65 nerve cells were grown and induced in 24 well plates for 2 days. Whole cell ROS and mitochondrial superoxide ROS were detected with 10 μM CM-H2DCFDA (Molecular Probes, C6827) and 5 μM MitoSox (Molecular Probes, M36008), respectively. Experiments were performed according to the manufacturer’s instructions and fluorescent measurements were read on a Spectramax M5 plate reader (Molecular Devices).

### Bioinformatics and statistics

Pathway analysis was performed using R package “WebGestaltR” [v0.3.1, https://academic.oup.com/nar/article/47/W1/W199/5494758] using GSEA method. The total list of genes/proteins were ranked by their (−log10 *p* value) × (sign of direction of change). Pathways with FDR < 0.1 were identified as significantly changed.

KEGG Mapper [https://www.kegg.jp/kegg/tool/map_pathway3.html] was used to visualize the integrated transcriptomic, metabolomics, and proteomic data. The logFC of the DE metabolites were shown in circles with color gradient representing the degree of change (red = up-regulated, blue = down-regulated). To integrate the transcriptomic and proteomic data, only the directions of change were considered. Red lines indicated up-regulation whereas blue lines indicated down-regulation for both transcriptomic and proteomic data. Purple lines indicated non-consistent directions of change between transcriptomic and proteomic data.

Principle Component Analysis (PCA) was performed by R function “prcomp” on the Metabolon normalized imputed values for metabolome, combat (from R package “sva” v3.26.0, https://bioconductor.org/packages/release/bioc/html/sva.html) batch corrected log-transformed normalized average spectrum values for proteome, and log-transformed FPKM values for transcriptome. Values were centered at zero for PCA and an ellipse were drawn to indicate the 95% confidence interval assuming a student distribution of the PCs. The top 500 most variable genes and proteins and total metabolites were used for PCA.

Association with subcellular compartments were tested using 1000 bootstrap. For each dataset, a same number of DE candidates were randomly drawn from reference total and the ratio of overlap with each subcellular compartments were calculated. A random seed of 10 was used for gene data and a random seed of 100 was used for protein data. Similar approach was used to calculate the significance of overlap ratio between Ferroptosis genes/proteins and subcellular compartments. A random seed of 12,345 was used for Ferroptosis gene data and a random seed of 42 was used for Ferroptosis protein data.

All statistics and figures were generated using R [R Core Team (2013). R: A language and environment for statistical computing. R Foundation for Statistical Computing, Vienna, Austria. URL http://www.R-project.org/] and R package “ggplot2” (https://cran.r-project.org/web/packages/ggplot2/index.html) unless mentioned specifically. Heatmaps were generated using R package “gplots” (https://cran.r-project.org/web/packages/gplots/index.html) on the row-wise *z*-scaled values used for PCA. Venn diagram was generated using R package “Venn Diagram” (https://cran.r-project.org/web/packages/VennDiagram/index.html).

RNA-Seq raw data were deposited at Gene Expression Omnibus with accession number GSE144194.

Where appropriate, experiments were performed at least three independent times. GraphPad Prism 8 was used for statistical analysis and exact *P* values are indicated (for *P* < 0.050).

## Supplementary information

TableS1

TableS2

TableS3

Figure S1
