# Supplementary figures and images for: Intracellular amyloid toxicity induces oxytosis/ferroptosis regulated cell death
Source: Cell Death Dis. 2020 Oct 6;11(10):828. doi: 10.1038/s41419-020-03020-9 (PMC7538552; doi:10.1038/s41419-020-03020-9)

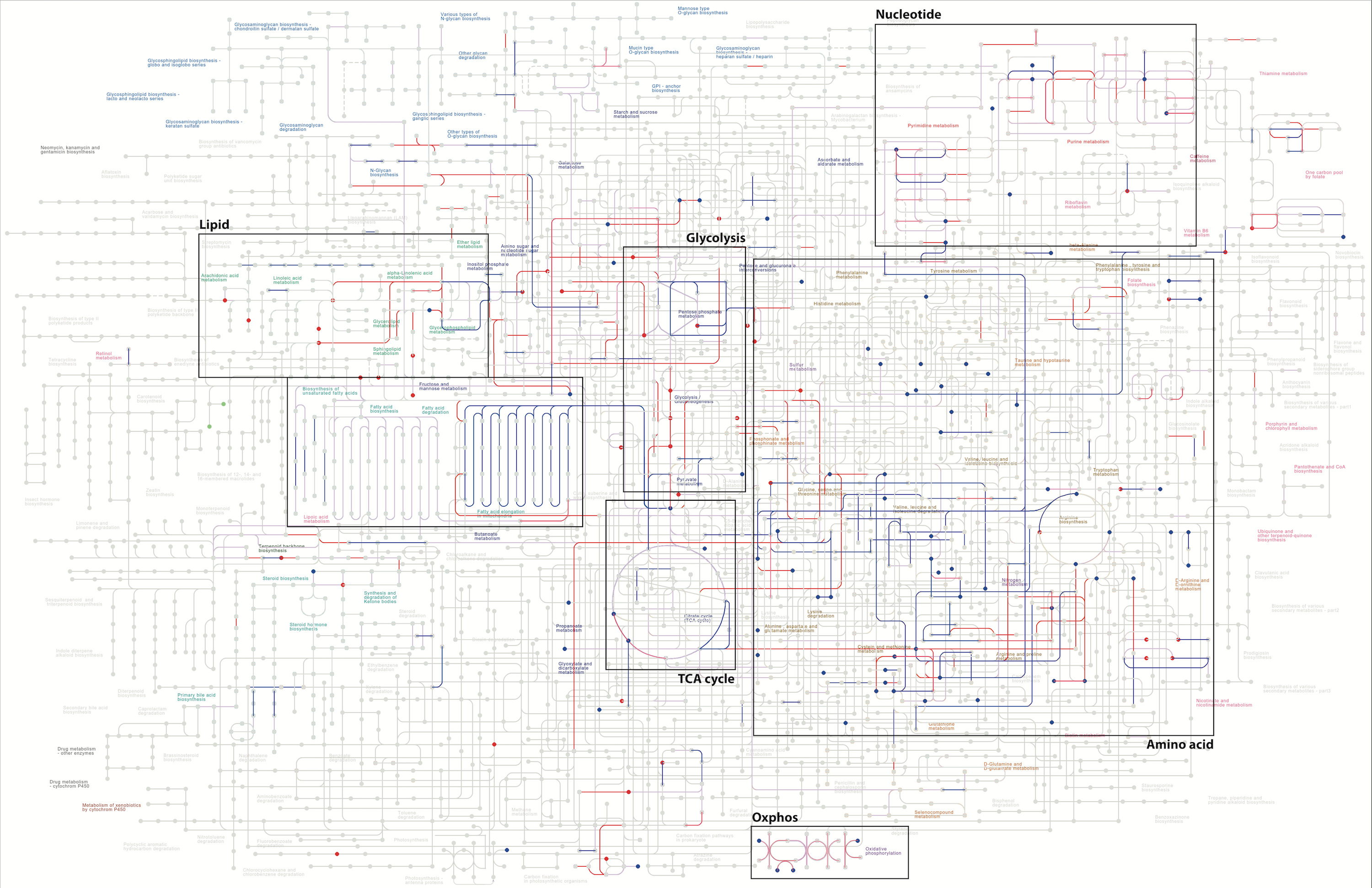

Supplement: Supplementary file 4 — Figure S1 [file 41419_2020_3020_MOESM4_ESM.tif]
